# Financial Satisfaction in Blended and Nuclear Families: A Dyadic Perspective

**DOI:** 10.21203/rs.3.rs-9476715/v1

**Published:** 2026-05-26

**Authors:** Mikel Van Cleve, Sonya Lutter

**Affiliations:** Texas Tech University; Texas Tech University

**Keywords:** actor–partner interdependence model, family structure, financial conflict, interdependent utility, kin selection

## Abstract

This study examines financial satisfaction among couples in blended and nuclear families using Waves 8–16 of the RAND Health and Retirement Study (2006–2022). Grounded in Becker’s interdependent utility theory and informed by Hamilton’s kin selection theory, this study integrates these frameworks to examine how differences in biological and relational ties may shape financial satisfaction within couples. Generalized structural equation models with ordered logistic regression were estimated within an actor–partner interdependence framework to examine both direct associations and cross-partner pathways consistent with interdependent utility. Across 16,050 household-wave observations from 7,421 households, respondents in blended families had 18.7% lower odds of being in a higher financial satisfaction category (OR = .813; 95% CI [.716, .923]) than those in otherwise similar nuclear families, with spouses showing a similar pattern. Mediation analyses revealed an asymmetric pathway: blended family status was negatively associated with spouse financial satisfaction, which, in turn, was associated with lower respondent financial satisfaction, whereas the reverse pathway was not statistically significant. These findings highlight how family structure and cross-partner dynamics shape couples’ financial satisfaction and underscore the importance of preference alignment and relational context in financial planning for blended families.

## Introduction

Family structure in the United States has changed substantially, with blended families (i.e., those including stepchildren) now representing a significant share of households. Estimates suggest that blended families comprise 26% of all marriages and 63% of remarriages (Pew Research Center, 2011; Stykes & Guzzo, 2015). Despite their prevalence, limited research has examined how blended family dynamics shape financial satisfaction. Financial satisfaction is an individual’s subjective appraisal of their financial condition, including perceptions of adequacy, stability, and the ability to meet current and future needs (Joo & Grable, 2004). It is closely linked to overall well-being and relationship satisfaction (Archuleta et al., 2011) and has also been associated with broader life satisfaction (Kruger et al., 2023; Matthew & Owusu, 2023).

Within couples, prior research shows that partners’ financial perceptions are interdependent and shaped by shared financial behaviors, roles, and coordination (Archuleta, 2013; Baryła-Matejczuk et al., 2020; Lee & Dustin, 2021). For example, lower financial integration and greater financial conflict are associated with poorer financial outcomes within couples (Lim & Morgan, 2021). However, this literature has largely treated families as homogeneous, overlooking differences between nuclear and blended family structures and providing limited insight into how variation in biological and relational ties may shape financial satisfaction within couples.

Blended families face distinct financial challenges due to complex relational dynamics and multiple subsystems (Antfolk et al., 2017). These dynamics can complicate financial decision-making and planning (Van Cleve & Klontz, 2022; Thornton, 2023). Such differences may reduce coordination in household financial decision-making and, in turn, financial satisfaction. Guided by interdependent utility theory (Becker, 1974) and kin selection theory (Hamilton, 1964), this study contributes to the literature by examining how differences in biological and relational ties may shape partners’ financial satisfaction and how these associations operate through cross-partner dynamics within couples.

### Theoretical Framework and Related Literature

Understanding differences in financial satisfaction across family structures requires a framework that accounts for both household utility and variation in biological and relational ties. Becker’s (1974) model of interdependent utility provides an economic framework for understanding financial transfers and joint household decision-making. In this model, an individual’s utility depends on both their own consumption and the welfare of other household members:

$${\text{U}}_{\text{A}}={\text{u}}_{\text{A}\left({\text{x}}_{\text{A}}\right)}+\sum_{\left\{i\neq A\right\}}\alpha_{\text{i}}{\text{u}}_{\text{i}\left({\text{x}}_{\text{i}}\right)}$$

where u_A_(x_A_) and u_i_(x_i_) are individual utility functions, and *α*_i_ > 0 represents the weight placed on the welfare of individual *i*. Couples achieve higher joint utility when these altruistic weights are aligned across family members, reflecting shared preferences over resource allocation. When weights differ, coordination may become more difficult, and financial decisions less efficient.

Kin selection theory provides an evolutionary framework for understanding variation in altruism in terms of genetic relatedness (Hamilton, 1964). The theory predicts that individuals allocate resources preferentially toward those with whom they share greater genetic ties, as reflected in Hamilton’s rule:

rB>C

where *C* is the cost to the donor, *B* is the benefit to the recipient, and *r* is the degree of genetic relatedness.

In family contexts, this implies that financial transfers may vary by biological relationship. Individuals may be more inclined to support biological children than stepchildren, for whom genetic relatedness is absent. Empirical evidence is consistent with this pattern, showing that stepparents tend to invest fewer resources in stepchildren than biological parents (Antfolk et al., 2017; Hirsch, 2021; Patterson et al., 2022). Applied within couples, kin selection theory suggests that altruistic weights may differ across partners, providing a potential source of preference heterogeneity in blended families.

Combined, interdependent utility theory and kin selection theory suggest that differences in biological and relational ties may produce variation in how partners weigh the well-being of family members. While interdependent utility theory emphasizes the importance of aligned preferences for efficient household decision-making, kin selection theory provides a mechanism through which these preferences may diverge. In blended families, such differences may introduce misalignment in these weights, increasing the likelihood of misaligned financial priorities and reduced coordination within couples, with implications for financial satisfaction.

### Financial Satisfaction

Prior research identifies several factors associated with financial satisfaction among couples. Shared financial goals and values are linked to higher financial satisfaction (Archuleta, 2013), while effective financial management behaviors, such as responsible credit use, budgeting, and timely bill payment, are associated with greater financial satisfaction and well-being (Baryła-Matejczuk et al., 2020; Kruger et al., 2023; Lee & Dustin, 2021). Financial coordination within couples also plays an important role. Dual-income couples who establish clear financial management rules report less conflict and higher financial satisfaction, even when maintaining separate finances (Lim & Morgan, 2021).

In contrast, poor coordination and financial management behaviors, such as inadequate savings, late payments, and insufficient insurance coverage, are associated with lower financial satisfaction (Baryła-Matejczuk et al., 2020; Lim & Morgan, 2021; Lee & Dustin, 2021). These patterns reflect variation in household financial efficiency, consistent with Becker’s (1974) interdependent utility framework, in which coordinated decision-making increases household utility while fragmented behaviors reduce it.

Among older adults, financial satisfaction tends to increase with age. This pattern has been linked to higher retirement savings and lower debt burdens in later life (Hansen et al., 2008; Hsieh, 2004). In this segment of the population, income and assets play a more limited role, with per capita income providing a more accurate predictor than raw income alone (Hsieh, 2004).

Although prior research highlights the importance of financial behaviors and economic resources, it has not examined how variation in altruistic preferences within couples, particularly those shaped by kinship differences, may influence financial satisfaction. This gap is particularly relevant in the context of blended families, where biological and step-relational ties may shape financial priorities and decision-making. Addressing this gap extends existing research by linking family structure to financial satisfaction through within-couple dynamics.

### Blended Family Dynamics

Research on family structure reveals meaningful differences between blended and nuclear families that can influence financial behavior. In nuclear families, both spouses share the same set of children, biological or legally adopted, creating a unified parental role and shared caregiving history. In contrast, blended families include children for whom only one spouse is the biological or adoptive parent, which can lead to differences in expectations, obligations, and relational closeness (Furstenberg et al., 2020).

These challenges may be more pronounced in later-life unions, where stepchildren are often adults and have not resided with the stepparent (Furstenberg et al., 2020).

Limited opportunities to build trust and establish shared routines may reduce both emotional and financial support in step-relationships. Weaker relational bonds are associated with lower expectations of intergenerational support and greater tension in areas such as estate planning (Van Cleve & Klontz, 2022). Although early work characterized blended families as less cohesive, more recent research emphasizes substantial variation across families, shaped by factors such as shared children, coparenting quality, and broader family dynamics (Sanner et al., 2021; Fang et al., 2025). However, ambiguity in family roles may increase the likelihood of conflict in financial decisions, including day-to-day money management and asset distribution.

These relational differences have direct implications for financial decision-making within couples. When partners hold different expectations regarding obligations to children and stepchildren, coordination may become more difficult. These differences are consistent with variation in how partners prioritize household financial obligations, which may increase financial conflict and contribute to lower financial satisfaction in blended families.

Although the literature lacks direct measures of financial satisfaction in blended families, prior research has examined financial behaviors within these households. Blended families are more likely to maintain separate bank accounts and manage finances independently (Eickmeyer et al., 2019; Raijas, 2011). They are also less likely to have both spouses employed (Eickmeyer et al., 2019), which may shape household resource flows and bargaining power. These characteristics may undermine marital unity and increase the risk of financial disagreements.

Money conflict is often more intense than other types of marital conflict and is associated with lower relationship quality (Britt & Huston, 2012; Dew et al., 2012; Peetz et al., 2023). Lower financial satisfaction has also been linked to financial conflict and related financial stressors (Lee & Dustin, 2021; Matthew & Owusu, 2023). Taken together, this literature suggests that the financial organization patterns more common in blended families may have implications for both relationship and financial outcomes, even though financial satisfaction has not been examined directly by family structure.

Blending biological and social ties also complicates resource allocation across children, stepchildren, and grandchildren. Parents must navigate competing demands shaped by age, need, and perceptions of fairness (Antfolk et al., 2017; Hirsch, 2021). These pressures may intensify when spouses prioritize their own children over stepchildren, as predicted by kin selection theory. Such differences are consistent with variation in how partners weigh the financial well-being of family members, which may complicate coordination in household financial decision-making. Additional obligations, such as alimony or child support to former spouses, may further strain household finances.

These dynamics are particularly relevant given the relationship between marital disruption and financial satisfaction. Divorced individuals report lower financial satisfaction (Fan & Babiarz, 2019), and blended families often form after divorce or widowhood (Silverberg, 2023). Among remarried couples, financial conflict appears to be an important mechanism linking economic strain to marital outcomes (Jackson et al., 2023).

For aging parents, stepchildren are less likely to assist stepparents, and support may be more conditional (Amorim, 2023; He, 2023; Patterson et al., 2022; Pew Research Center, 2011; Van der Pas & van Tilburg, 2025; van Houdt et al., 2020). For example, 85% of biological children report feeling responsible for helping aging parents compared with 56% of stepchildren (Pew Research Center, 2011). These differences in expected and realized support further underscore why financial satisfaction may differ in later-life blended families.

### Hypotheses

Family structure (nuclear vs. blended) serves as a proxy for potential differences in couples’ interdependent utility weighting across partners with respect to children and stepchildren. Financial satisfaction for both the respondent and spouse captures each partner’s subjective evaluation of their financial situation, providing observable outcomes through which aligned or misaligned preferences may be reflected. Within a dyadic framework, one partner’s financial satisfaction may also influence the other’s, reflecting interdependence in household utility (Becker, 1974). The integrated theoretical framework anticipates both direct differences in financial satisfaction between nuclear and blended families and cross-spousal mediation effects consistent with interdependent utility theory, as outlined in the following hypotheses.

- **H1a**: Respondents in blended families will report lower financial satisfaction than respondents in nuclear families.
- **H1b**: Spouses in blended families will report lower financial satisfaction than spouses in nuclear families.
- **H2a**: The spouse’s financial satisfaction will mediate the relationship between family structure and the respondent’s financial satisfaction.
- **H2b**: The respondent’s financial satisfaction will mediate the relationship between family structure and the spouse’s financial satisfaction.

## Methods

### Data

This study used quantitative secondary data from the RAND Health and Retirement Study (HRS) Longitudinal and Family files. The HRS is a nationally representative panel study of U.S. adults aged 50 and older, sponsored by the National Institute on Aging (grant numbers NIA U01AG009740 and NIA R01AG073289) and conducted by the University of Michigan (Health and Retirement Study 2025a, 2025b). Data from Waves 8–16 (2006–2022) were used, as these waves include the Leave Behind Psychosocial Questionnaire (LBQ), which includes a measure of financial satisfaction. The RAND HRS LBQ weight was applied in descriptive analyses to ensure national representativeness. The RAND HRS captures a population in which blended families are increasingly common due to later-life divorce and remarriage, and where financial decision-making often involves established assets and complex intergenerational ties. These features make it a particularly informative context for examining how family structure shapes financial satisfaction.

### Sample

After merging files, the sample included 407,106 household-wave observations across 45,234 unique households. The analytic sample was restricted to couple-households with at least one child (92,323 observations; 21,053 households). To avoid duplicate household-wave records, the sample was further limited to one observation per household per wave, resulting in 46,180 observations across 11,165 households. Attrition among LBQ participants reduced the sample to 17,431 observations across 8,075 households. The sample was then restricted to households with consistent family structure across waves to facilitate between-group comparisons, yielding 16,126 observations across 7,464 households.

Complete case analysis produced a final analytic sample of 16,050 household-wave observations from 7,421 households. The designated primary respondent is referred to as the respondent, and their partner is referred to as the spouse. In nearly all cases, the respondent also served as the household’s financial respondent.

### Measures

Household income and wealth were adjusted to 2022 dollars using the Consumer Price Index for All Urban Consumers (CPI-U) annual averages from the U.S. Bureau of Labor Statistics (2024). Wave-specific adjustments were applied to ensure comparability across time. Income and wealth were then transformed using the inverse hyperbolic sine (IHS) function to address skewness while accommodating zero and negative values (Aihounton & Henningsen, 2019; Friedline et al., 2015).

### Dependent Variable

Financial satisfaction was measured on a five-point Likert scale for both the respondent and their spouse, ranging from 1 (not at all satisfied) to 5 (completely satisfied).

### Independent Variable

The primary independent variable was family structure, operationalized as a binary indicator: nuclear family (0) versus blended family (1). A household was classified as nuclear if neither partner reported stepchildren and at least one reported own children, and as blended if either partner reported at least one stepchild; households without children were excluded. The American Psychological Association (2018) defines nuclear families as those including biological or legally adopted children. In the RAND HRS data, adopted children are coded as “own children,” and adoption status is not consistently available after 1992 (RAND HRS support team, personal communication, November 2025); accordingly, “own children” include both biological and adopted children.

### Mediating Variable

The mediating variable was partner financial satisfaction. Two mediation models were estimated to capture dyadic effects. In the first, the spouse’s financial satisfaction mediated the relationship between family structure and the respondent’s financial satisfaction. In the second, the respondent’s financial satisfaction mediated the relationship between family structure and the spouse’s financial satisfaction.

### Control Variables

Control variables were included to account for potential confounding in the relationship between family structure and financial satisfaction. Individual-level controls for both partners included age, gender, race, marital status, health, and educational attainment. Health was reverse-coded to align with the ordering of other categorical variables. Household-level controls included the number of residents, as well as household income and wealth (net worth). Wave indicators were included to account for period effects.

### Analysis

Generalized structural equation models (GSEM) with ordered logistic regression were estimated in Stata 19.5. Models were estimated without survey weights because the analytic sample is a restricted subset of HRS households, whereas RAND HRS weights are designed for full-sample population inference. Survey-weighted models were also evaluated as a robustness check.

Given the dyadic and longitudinal structure of the data, standard errors were clustered at the household level, and a household-level random intercept was included to account for unobserved heterogeneity across waves. Mundlak-corrected specifications incorporated the means of time-varying covariates to address potential correlation between predictors and unobserved effects (Mundlak, 1978). Because family structure may reflect selection processes, results are interpreted as associations rather than causal effects.

### Dyadic Statistical Framework

**Figure 1:**
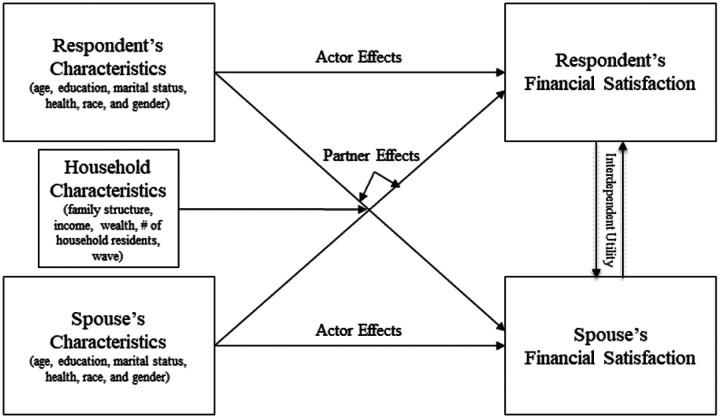
The Actor-Partner Interdependence Model (APIM) of Financial Satisfaction *Notes:* Actor effects represent the influence of an individual’s characteristics on their own financial satisfaction, while partner effects represent the influence of a partner’s characteristics on the individual’s financial satisfaction. Marital status, race, and gender were excluded from partner effects due to multicollinearity.

An actor–partner interdependence model (APIM) (Loeys et al., 2014) was used to estimate dyadic relationships between family structure and financial satisfaction for respondents and their spouses. As shown in Fig. 1, each partner’s financial satisfaction is modeled as a function of their own characteristics (actor effects), their partner’s characteristics (partner effects), and household-level characteristics. These characteristics capture demographic, socioeconomic, and health-related factors associated with household financial well-being. The bidirectional association between partners’ financial satisfaction reflects interdependence in which each partner’s outcomes are associated with the other’s well-being, consistent with interdependent utility theory. Family structure (nuclear vs. blended) serves as a between-household characteristic that influences both partners’ financial satisfaction and the cross-spousal pathways examined in the mediation models.

#### Notes

Actor effects represent the influence of an individual’s characteristics on their own financial satisfaction, while partner effects represent the influence of a partner’s characteristics on the individual’s financial satisfaction. Marital status, race, and gender were excluded from partner effects due to multicollinearity.

### Model 1

Models 1a and 1b estimate the respondent’s and spouse’s financial satisfaction, respectively, as a function of family structure (blended vs. nuclear), controlling for individual- and household-level characteristics. These models exclude cross-spousal outcome pathways (i.e., one partner’s financial satisfaction predicting the other’s) but incorporate dyadic structure through actor- and partner-level covariates. Model 1 thus provides a baseline estimate of the association between family structure and each partner’s financial satisfaction prior to introducing mediation.

### Model 2

**Figure 2:**
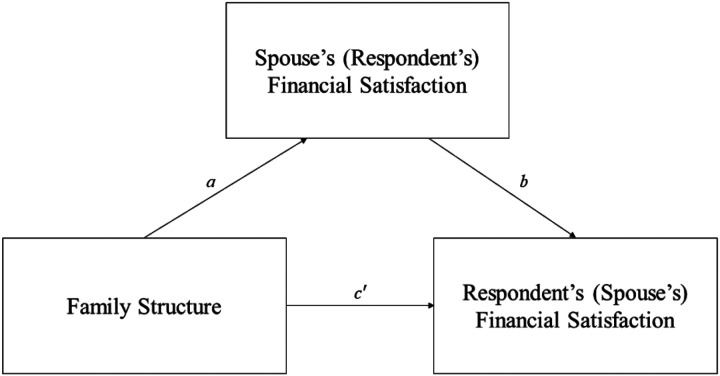
Mediating Effect of Spouse (Respondent) Financial Satisfaction (Model 2) *Notes:* Path *a* represents the effect of family structure on spouse (respondent) financial satisfaction; path *b* represents the effect of spouse (respondent) financial satisfaction on respondent (spouse) financial satisfaction; and path *c′* represents the direct effect of family structure on respondent (spouse) financial satisfaction. The indirect effect is given by *a* × *b*.

Model 2 evaluates mediation using GSEM by specifying cross-spousal pathways between family structure and financial satisfaction. In Model 2a, spouse financial satisfaction is modeled as a function of family structure, and both spouse satisfaction and family structure predict respondent financial satisfaction. Model 2b reverses this structure, with respondent financial satisfaction serving as the mediator. Both models include the same demographic, financial, and household-level covariates as Models 1a and 1b. Figure 2 illustrates the mediation pathways.

#### Notes

Path *a* represents the effect of family structure on spouse (respondent) financial satisfaction; path *b* represents the effect of spouse (respondent) financial satisfaction on respondent (spouse) financial satisfaction; and path *c′* represents the direct effect of family structure on respondent (spouse) financial satisfaction. The indirect effect is given by *a* × *b*.

### Post-Estimation Procedures

For Models 1a and 1b, odds ratios were derived from GSEM estimates to assess the likelihood of being in a higher financial satisfaction category by family structure. For Models 2a and 2b, indirect effects and total effects were tested within the GSEM framework, and coefficients were exponentiated to present odds ratios consistent with the direct effects.

### Robustness Checks

Weighted regressions using the RAND HRS LBQ weight were conducted as a robustness check. Because GSEM ordered logit models with household random intercepts cannot be estimated with probability weights, weighted models were estimated using pooled GSEM ordered logit specifications with the same covariates and pathways as the primary models and standard errors clustered at the household level. To assess sensitivity to family classification, supplemental models were stratified by parental structure. In addition, given evidence that dyadic effects may differ by gender (Carr et al., 2016; Brauer et al., 2021), models were estimated separately for men and women.

### Model Fit Diagnostics

Because GSEM differs from traditional structural equation modeling, conventional fit indices (e.g., CFI, RMSEA, TLI) are not available in Stata (StataCorp, 2025). Model adequacy was therefore evaluated using alternative diagnostics. Adjusted Wald tests assessed the joint significance of family structure and covariates, and likelihood ratio (LR) tests compared each full model with a nested constant-only model to evaluate improvements in model fit.

## Results

### Descriptive Statistics

Table 1 presents weighted descriptive statistics for nuclear and blended families in 2006 and 2022, providing a comparison of the first and last waves used in the analyses. Nuclear families comprised 67.6% of the sample in 2006, declining to 61.7% in 2022, while blended families increased from 32.4% to 38.3%, reflecting their growing prevalence among older adults. Within blended families, the distribution of which partner was in the stepparent role shifted modestly over time, with respondent-only increasing (19.8% to 25.8%), spouse-only decreasing (33.5% to 27.1%), and both partners remaining relatively stable (46.7% to 47.1%).

In both waves, respondents and spouses in blended families reported lower financial satisfaction than those in nuclear families, with greater concentration in the lower satisfaction categories. In 2006, 23.9% of respondents in blended families reported being “not at all” or “not very” satisfied, compared to 17.3% in nuclear families. By 2022, high financial satisfaction (“very” or “completely” satisfied) increased substantially among nuclear families (71.7%), while remaining lower among blended families (51.8%). Spouse financial satisfaction followed a similar pattern.

### Trends in Financial Satisfaction

**Figure 3:**
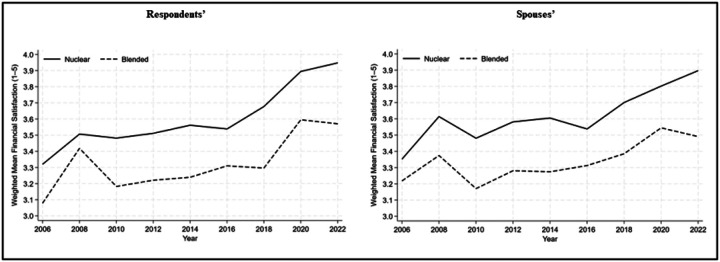
Survey-Weighted Mean Financial Satisfaction for Respondents’ and Spouses’ (2006–2022) *Source*: RAND HRS, Waves 8–16. *Notes*: The HRS introduced a new cohort, early Generation X, in 2022 (Health and Retirement Study, 2026).

Figure 3 shows that respondents’ and spouses’ mean financial satisfaction increased over time for both family structures, with nuclear families consistently reporting higher levels across all waves. Although both groups experienced gains, the gap between nuclear and blended families persisted throughout the period.

#### Source

RAND HRS, Waves 8–16.

#### Notes

The HRS introduced a new cohort, early Generation X, in 2022 (Health and Retirement Study, 2026).

### Multivariate Results

Odds ratios from each analysis for the relationship between family structure and financial satisfaction for both respondents and their spouses are presented below. Robustness checks produced results that were generally consistent in direction and magnitude across specifications.

### Model 1: The Relationship Between Family Structure and Financial Satisfaction

#### Direct Effects of Family Structure.

Respondents in blended families reported lower financial satisfaction than those in otherwise similar nuclear families, with 18.7% lower odds of being in a higher satisfaction category (OR = .813; 95% CI [.716, .923]; Table 2). Spouses showed a similar pattern, with 13.7% lower odds of higher satisfaction (OR = .863; 95% CI [.759, .982]; Table 3).

### Model 2: The Mediating Effect of Partner Financial Satisfaction

#### Spouse → Respondent Pathway.

Blended family status was indirectly associated with 11.2% lower odds of respondents reporting higher financial satisfaction through the spouse’s financial satisfaction (indirect OR = .888, 95% CI [.815, .962]), as shown in Table 4. The direct effect was not statistically significant, while the total effect remained significant, indicating that the association operates primarily through the spouse’s financial satisfaction.

#### Respondent → Spouse Pathway.

When respondent financial satisfaction was specified as the mediator, the indirect effect was not statistically significant (Table 5). However, both the direct and total effects remained statistically significant. These results indicate that blended family status does not operate through respondents’ financial satisfaction to influence their spouses’ financial satisfaction.

## Discussion

The results reveal consistent, robust differences in financial satisfaction across family structures. Findings are interpreted in relation to interdependent utility theory and kin selection theory, which together provide a framework for understanding how relational and biological ties shape financial outcomes.

### Hypotheses 1a and 1b: Financial Satisfaction Across Family Structures

Across Models 1a and 1b, respondents and spouses in blended families had significantly lower odds of higher financial satisfaction than those in otherwise similar nuclear families. These associations remained stable after adjusting for demographic characteristics, income, wealth, and health, and were consistent across weighted, gender-stratified, and parental-structure specifications.

These findings are consistent with interdependent utility theory (Becker, 1974), which posits that household decision-making depends on the extent to which partners internalize one another’s welfare. When partners assign similar weights to the same family members, financial decisions are more coordinated and efficient. In nuclear families, shared parental roles may facilitate alignment in financial priorities, whereas in blended families, distinct kinship ties may increase the likelihood of divergent preferences. Such differences may contribute to misaligned financial priorities regarding saving, spending, and transfers (Antfolk et al., 2017; Hirsch, 2021; Raijas, 2011).

Kin selection theory (Hamilton, 1964) further contextualizes these results by highlighting the role of genetic relatedness in shaping altruistic behavior. Lower relatedness in step-relationships is associated with weaker perceived obligations and lower expectations of support (Patterson et al., 2022; van Houdt et al., 2020). These structural differences provide a plausible mechanism for the lower financial satisfaction observed in blended families.

### Hypotheses 2a and 2b: Partner Mediation

The mediation analyses revealed a clear and asymmetric pattern. Only the spouse-to-respondent pathway was statistically significant. Blended family status was associated with lower spouse financial satisfaction, which in turn was associated with lower respondent financial satisfaction. The respondent-to-spouse pathway was not significant, supporting H2a but not H2b. This asymmetry remained consistent across all robustness checks.

The significant spouse-to-respondent pathway is consistent with asymmetric internalization of utility. The respondent’s financial satisfaction is more closely tied to the spouse’s satisfaction than vice versa. Interdependent utility theory allows for unequal weighting of partner welfare and predicts spillover when one partner’s utility more strongly influences the other’s preferences (Becker, 1974).

Kin selection theory provides additional context (Hamilton, 1964). Differences in kinship ties across partners may shape how financial outcomes are transmitted within couples, as stepparent–stepchild relationships tend to involve weaker expectations of support, greater relational uncertainty, and lower perceived obligation (Patterson et al., 2022; van Houdt et al., 2020). These dynamics are consistent with the observed mediation from spouse to respondent. However, patterns across parental configurations provide only limited support for this interpretation. When the spouse was in the stepparent role, the indirect effect remained negative and statistically significant, whereas when only the respondent was the stepparent, it was not.

### Implications for Practice

These findings have clear implications for financial planners, counselors, and professionals working with blended families. These families are associated with lower financial satisfaction, suggesting a need for more proactive guidance on financial coordination, communication, and expectation-setting. Prior research shows that blended families are more likely to maintain separate accounts and fragmented financial systems, which can complicate coordination and increase financial conflict (Eickmeyer et al., 2019; Lim & Morgan, 2021; Raijas, 2011). Supporting couples in developing shared financial goals and clearly defined roles may help reduce misaligned preferences. The asymmetric mediation results further highlight the importance of engaging both partners in the planning process. Because the spouse’s financial satisfaction influences the respondent’s satisfaction but not vice versa, practitioners should not assume that one partner’s satisfaction equals the household’s.

Practitioners can also draw on tools from financial psychology to facilitate more effective conversations. Techniques such as behavioral rehearsal exercises (e.g., the Van Cleve–Klontz Role Play Model for Couples) encourage active listening and mutual understanding (Van Cleve & Klontz, 2022). Genograms may also be used to map family relationships and highlight biological, step-, and emotional ties. Making these dynamics explicit can support more productive discussions about financial roles, expectations, and long-term planning.

### Limitations and Future Research

Several limitations should be noted. First, the sample includes only adults aged 50 and older. This focus aligns with the typical financial planning client (Northwestern Mutual, 2024), but may limit generalizability to younger populations. Second, financial satisfaction is a subjective measure and may reflect emotional or relational factors not fully captured by observed financial variables. Third, although the use of longitudinal data and Mundlak corrections strengthens inference, the results remain associative rather than causal, as family structure is not strictly exogenous.

Future research could examine how specific financial behaviors, such as account integration, budgeting, and financial secrecy, influence financial satisfaction. Additional work is also needed to better understand the individual- and family-level processes underlying the formation of blended families. In particular, examining variation across racial and socioeconomic subgroups may clarify how early-life experiences and structural inequalities intersect with family structure to shape financial outcomes.

## Conclusion

Blended families represent a growing share of U.S. households and face distinct structural and relational challenges that may affect financial satisfaction. This study provides evidence that blended family status is associated with lower odds of higher financial satisfaction for both partners relative to otherwise similar nuclear families, with differences operating in part through cross-partner dynamics grounded in interdependent utility and kin selection. By identifying both direct and mediated pathways linking family structure to financial satisfaction, these findings extend the literature and improve understanding of financial satisfaction among couples, while highlighting the importance of relational context in shaping financial outcomes and guiding effective financial planning in blended families.

## Figures and Tables

**Table 1:** Weighted Descriptive Statistics for 2006 and 2022

	2006			2022		
	Nuclear	Blended	Overall	Nuclear	Blended	Overall
	(n = 1,574)	(n = 786)	(n = 2,360)	(n = 585)	(n = 455)	(n = 1,040)
**Family Structure (%)**	67.6%	32.4%	100.0%	61.7%	38.3%	100.0%
**Number of Household Residents**	2.62	2.45	2.56	2.49	2.53	2.51
**Respondent’s Age**	65.34	63.37	64.70	69.07	67.76	68.57
**Spouse’s Age**	64.35	59.32	62.72	68.59	64.92	67.19
**Median Household Income (Real $)**	85,684	86,396	86,304	94,520	71,972	85,212
**Median Household Wealth (Real $)**	472,661	370,174	445,660	710,000	437,500	622,000
**Parental Structure**						
**Shared Children Only**	100.0%	-	67.6%	100.0%	-	61.7%
**R Has Stepchildren**	-	19.8%	6.4%	-	25.8%	9.9%
**S Has Stepchildren**	-	33.5%	10.8%	-	27.1%	10.4%
**Both Have Stepchildren**	-	46.7%	15.1%	-	47.1%	18.0%
**Respondent Education Level**						
**< High School**	11.3%	13.2%	11.9%	5.4%	8.8%	6.7%
**GED**	4.1%	6.6%	4.9%	3.9%	4.2%	4.0%
**HS Diploma**	28.7%	28.5%	28.7%	22.8%	22.1%	22.6%
**Some College**	22.6%	25.3%	23.4%	18.6%	30.1%	23.0%
**College +**	33.3%	26.4%	31.1%	49.3%	34.7%	43.7%
**Spouse Education Level**						
**< High School**	16.7%	20.0%	17.7%	7.7%	7.9%	7.8%
**GED**	3.8%	4.6%	4.1%	2.0%	7.6%	4.2%
**HS Diploma**	34.2%	28.2%	32.2%	25.7%	24.2%	25.1%
**Some College**	23.2%	27.0%	24.4%	25.8%	33.8%	28.9%
**College +**	22.1%	20.2%	21.5%	38.7%	26.6%	34.1%
**Respondent Gender**						
**Male**	64.7%	63.4%	64.3%	65.8%	59.5%	63.4%
**Female**	35.3%	36.6%	35.7%	34.2%	40.5%	36.6%
**Spouse Gender**						
**Male**	35.1%	36.5%	35.6%	33.2%	40.8%	36.1%
**Female**	64.9%	63.5%	64.4%	66.8%	59.2%	63.9%
**Respondent Race**						
**White**	92.2%	84.9%	89.8%	87.3%	80.4%	84.6%
**Black**	3.4%	10.5%	5.7%	2.6%	11.6%	6.0%
**Other**	4.4%	4.6%	4.5%	10.1%	8.0%	9.3%
**Spouse Race**						
**White**	91.6%	83.7%	89.1%	87.9%	80.3%	85.0%
**Black**	3.6%	10.4%	5.8%	3.2%	11.6%	6.4%
**Other**	4.8%	5.9%	5.1%	8.9%	8.1%	8.6%
**Respondent Marital Status**						
**Married**	98.8%	91.7%	96.5%	98.7%	90.5%	95.5%
**Married, Sp Absent**	0.5%	0.9%	0.6%	0.3%	1.1%	0.6%
**Partnered**	0.7%	7.4%	2.9%	1.0%	8.4%	3.8%
**Spouse Marital Status**						
**Married**	98.8%	91.5%	96.4%	98.7%	91.0%	95.7%
**Married, Sp Absent**	0.5%	0.9%	0.6%	0.3%	1.1%	0.6%
**Partnered**	0.7%	7.5%	2.9%	1.0%	7.9%	3.6%
**Respondent Self-Rated Health**						
**Poor**	4.8%	8.4%	6.0%	2.8%	3.8%	3.2%
**Fair**	15.1%	19.7%	16.6%	12.7%	15.7%	13.9%
**Good**	30.5%	27.1%	29.4%	29.1%	35.8%	31.7%
**Very Good**	35.8%	29.9%	33.9%	43.4%	36.6%	40.8%
**Excellent**	13.7%	14.8%	14.1%	12.0%	8.0%	10.5%
**Spouse Self-Rated Health**						
**Poor**	5.3%	5.0%	5.2%	2.8%	5.1%	3.7%
**Fair**	15.0%	17.9%	16.0%	11.2%	19.3%	14.3%
**Good**	32.4%	29.1%	31.3%	30.5%	34.5%	32.0%
**Very Good**	34.6%	31.8%	33.7%	46.0%	33.5%	41.2%
**Excellent**	12.6%	16.2%	13.8%	9.6%	7.6%	8.8%
**Respondent Financial Satisfaction**						
**Not At All Satisfied**	5.6%	10.7%	7.3%	0.5%	2.3%	1.2%
**Not Very Satisfied**	11.7%	13.2%	12.2%	3.3%	13.0%	7.0%
**Somewhat Satisfied**	38.4%	41.4%	39.4%	24.4%	32.9%	27.7%
**Very Satisfied**	33.8%	27.0%	31.6%	44.4%	28.9%	38.5%
**Completely Satisfied**	10.6%	7.7%	9.7%	27.3%	22.9%	25.6%
**Spouse Financial Satisfaction**						
**Not At All Satisfied**	4.6%	7.9%	5.6%	1.3%	4.2%	2.4%
**Not Very Satisfied**	12.3%	13.2%	12.6%	5.4%	10.7%	7.4%
**Somewhat Satisfied**	38.9%	38.8%	38.9%	24.3%	35.2%	28.5%
**Very Satisfied**	31.8%	29.4%	31.0%	40.4%	31.7%	37.1%
**Completely Satisfied**	12.4%	10.7%	11.9%	28.6%	18.3%	24.6%

Source

RAND HRS, Waves 8 and 16.

Notes

Percentages may not equal 100% due to rounding. All monetary values are adjusted to 2022 dollars using the CPI-U annual averages from the U.S. Bureau of Labor Statistics.

**Table 2:** Odds Ratios for the Relationship Between Family Structure and the Respondent’s Financial Satisfaction (Model 1a)

Respondent’s Financial Satisfaction	Household-Level Effects					
	OR	SE	95% CI			
**Family Structure (Nuclear)**						
**Blended**	813***	.053	[.716, .923]			
**Number of Household Residents**	.946	.034	[.883, 1.014]			
**Household Income**	1115***	.034	[1.051, 1.183]			
**Household Wealth**	1.033***	.008	[1.018, 1.048]			
**Wave (2006)**						
**2008**	2.076***	.172	[1.764, 2.442]			
**2010**	1 671***	.117	[1.456, 1.917]			
**2012**	1.804***	.162	[1.513, 2.152]			
**2014**	1 771***	.160	[1.483, 2.114]			
**2016**	1.753***	.194	[1.411, 2.177]			
**2018**	2 120***	.253	[1.677, 2.680]			
**2020**	3.350***	.469	[2.546, 4.408]			
**2022**	2.857***	.431	[2.126, 3.840]			
	Actor Effects			Partner Effects		
	OR	SE	95% CI	OR	SE	95% CI
**Education (< High School)**						
**GED**	.500***	.082	[.362, .691]	.866	.132	[.643, 1.168]
**Family Structure (Nuclear)**						
**HS Diploma**	.698**	.081	[.556, .876]	.995	.101	[.816, 1.213]
**Some College**	597***	.073	[.470, .759]	.968	.105	[.782, 1.197]
**College +**	.707**	.092	[.548, .913]	1.382**	.166	[1.091, 1.749]
**Self-Rated Health (Poor)**						
**Fair**	1.109	.181	[.805, 1.528]	1.164	.165	[.883, 1.536]
**Good**	1.380	.235	[.989, 1.926]	1.398*	.203	[1.051, 1.859]
**Very Good**	1.615**	.284	[1.144, 2.281]	1.401*	.212	[1.042, 1.884]
**Excellent**	1.654**	.325	[1.125, 2.431]	1.632**	.279	[1.167, 2.283]
**Race (White)**						
**Black**	.668	.172	[.403, 1.106]	.912	.234	[.551, 1.508]
**Other**	1.127	.154	[.862, 1.472]	1.201	.157	[.929, 1.552]
**Gender (Male)**						
**Female**	1.005	.064	[.887, 1.140]	-	-	-
**Marital Status (Married)**						
**Married, Spouse Absent**	.789	.237	[.438, 1.420]	-	-	-
**Partnered**	.563	.203	[.279, 1.140]	-	-	-
**Age**	1.052***	.011	[1.031, 1.073]	-	-	-

RAND HRS, Waves 8–16.

Notes

Base category in parentheses. OR = odds ratios; SE = standard errors. Race and gender were excluded from partner effects due to multicollinearity; household-level variables do not have partner effects. Means for the time-varying covariates (used for the Mundlak corrections) were computed and can be provided upon request.

*GSEM Model Fit*: Adjusted Wald Test χ^2^ = 2349.27; *p* < .001; Likelihood Ratio Test: χ^2^ = 2794.21; *p* < .001

*** *p* < .001,

** *p* < .01

* *p* ≤ .05

**Table 3:** Odds Ratios for the Relationship Between Family Structure and the Spouse’s Financial Satisfaction (Model 1b)

Spouse’s Financial Satisfaction	Household-Level Effects					
	OR	SE	95% CI			
**Family Structure (Nuclear)**						
**Blended**	.863*	.057	[.759, .982]			
**Number of Household Residents**	.887***	.033	[.824, .954]			
**Household Income**	1.064*	.032	[1.004, 1.129]			
**Household Wealth**	1.029***	.007	[1.015, 1.043]			
**Wave (2006)**						
**2008**	1.898	.158	[1.613, 2.234]			
**2010**	1.412	.100	[1.229, 1.621]			
**2012**	1.680	.148	[1.413, 1.998]			
**2014**	1.676	.153	[1.401, 2.005]			
**2016**	1.502	.164	[1.212, 1.861]			
**2018**	1.806	.216	[1.429, 2.283]			
**2020**	2.574	.352	[1.970, 3.365]			
**2022**	2.417	.357	[1.809, 3.229]			
Actor Effects	Partner Effects					
	OR	SE	95% CI	OR	SE	95% CI
**Education (< High School)**						
**GED**	.708	.111	[.521, .963]	.793	.130	[.575, 1.094]
**HS Diploma**	.947	.098	[.774, 1.159]	.895	.101	[.718, 1.115]
**Family Structure (Nuclear)**						
**Some College**	.806	.088	[.650, 1.000]	.678	.081	[.537, .856]
**College +**	.970	.117	[.766, 1.228]	.937	.118	
**Self-Rated Health (Poor)**						
**Fair**	.789	.135	[.564, 1.102]	1.236	.181	[.927, 1.647]
**Good**	1.041	.181	[.740, 1.465]	1.460	.219	[1.088, 1.960]
**Very Good**	1.172	.215	[.818, 1.679]	1.636	.260	[1.199, 2.233]
**Excellent**	1.171	.236	[.789, 1.738]	1.789	.320	[1.260, 2.539]
**Race (White)**						
**Black**	.597	.143	[.374, .954]	.942	.224	[.592, 1.501]
**Other**	1.177	.153	[.912, 1.520]	1.208	.167	[.921, 1.584]
**Gender (Male)**						
**Female**	1.362	.086	[1.203, 1.542]	-	-	-
**Marital Status (Married)**						
**Married, Spouse Absent**	.693	.212	[.380, 1.264]	-	-	-
**Partnered**	.427*	.178	[.188, .969]	-	-	-
**Age**	1.041***	.010	[1.021, 1.061]	-	-	-

Source

RAND HRS, Waves 8–16.

Notes

Base category in parentheses. OR = odds ratios; SE = standard errors. Race and gender were excluded from partner effects due to multicollinearity; household-level variables do not have partner effects. Means for the time-varying covariates (used for the Mundlak corrections) were computed and can be provided upon request.

*GSEM Model Fit*: Adjusted Wald Test χ^2^ = 2128.44; *p* < .001; Likelihood Ratio Test: χ^2^ = 2463.34; *p* < .001

*** *p* < .001,

** *p* < .01

* *p* ≤ .05

**Table 4:** Odds Ratios for the Mediating Effect of the Spouse’s Financial Satisfaction on the Relationship Between Family Structure and the Respondent’s Financial Satisfaction (Model 2a)

Mediation Pathway	Direct Effect			Indirect Effect			Total Effect		
	OR	SE	95% CI	OR	SE	95% CI	OR	SE	95% CI
Blended Family→ Spouse’s Fin Sat→ Respondent’s Fin Sat	.934	.059	[.825, 1.057]	.888**	.038	[.815, .962]	.829*	.062	[.707, .951]

Source

RAND HRS, Waves 8–16.

Notes

OR = odds ratios; SE = standard errors; Fin Sat = financial satisfaction.

*GSEM Model Fit*: Adjusted Wald Test χ^2^ = 6579.05; *p* < .001; Likelihood Ratio Test: χ^2^ = 7033.74; *p* < .001

*** *p* < .001,

** *p* < .01

* *p* ≤ .05

**Table 5:** Odds Ratios for the Mediating Effect of the Respondent’s Financial Satisfaction on the Relationship Between Family Structure and the Spouse’s Financial Satisfaction (Model 2b)

Mediation Pathway	Direct Effect			Indirect Effect			Total Effect		
	OR	SE	95% CI	OR	SE	95% CI	OR	SE	95% CI
Blended Family→ Respondent’s Fin Sat→ Spouse’s Fin Sat	.880*	.056	[.776, .996]	.944	.038	[.869, 1.019]	.830*	.062	[.708, .952]

Source: RAND HRS, Waves 8–16.

Notes: OR = odds ratios; SE = standard errors.

*GSEM Model Fit*: Adjusted Wald Test χ^2^ = 7007.25; *p* < .001; Likelihood Ratio Test: χ^2^ = 7223.09; *p* < .001

*** p < .001,

** p < .01

* p ≤ .05

## Data Availability

The data that support the findings of this study are publicly available from the Health and Retirement Study (HRS), produced and distributed by the University of Michigan with funding from the National Institute on Aging. RAND HRS data files are available at https://hrs.isr.umich.edu/data-products and https://www.rand.org/health/surveys/hrs.html. Data are subject to the terms and conditions of use established by the HRS.
